# Biocide Exposure Induces Changes in Susceptibility, Pathogenicity, and Biofilm Formation in Uropathogenic *Escherichia coli*

**DOI:** 10.1128/AAC.01892-18

**Published:** 2019-02-26

**Authors:** E. L. Henly, J. A. R. Dowling, J. B. Maingay, M. M. Lacey, T. J. Smith, S. Forbes

**Affiliations:** a Biomolecular Sciences Research Centre, Sheffield Hallam University, Sheffield, United Kingdom

**Keywords:** biocide, biofilm, susceptibility

## Abstract

Uropathogenic Escherichia coli (UPEC) is a frequent cause of catheter-associated urinary tract infection (CAUTI). Biocides have been incorporated into catheter coatings to inhibit bacterial colonization while, ideally, exhibiting low cytotoxicity and mitigating the selection of resistant bacterial populations. We compared the effects of long-term biocide exposure on susceptibility, biofilm formation, and relative pathogenicity in eight UPEC isolates. MICs, minimum bactericidal concentrations (MBCs), minimum biofilm eradication concentrations (MBECs), and antibiotic susceptibilities were determined before and after long-term exposure to triclosan, polyhexamethylene biguanide (PHMB), benzalkonium chloride (BAC), and silver nitrate. Biofilm formation was quantified using a crystal violet assay, and relative pathogenicity was assessed via a Galleria mellonella waxworm model. Cytotoxicity and the resulting biocompatibility index values were determined by use of an L929 murine fibroblast cell line. Biocide exposure resulted in multiple decreases in biocide susceptibility in planktonic and biofilm-associated UPEC. Triclosan exposure induced the largest frequency and magnitude of susceptibility decreases at the MIC, MBC, and MBEC, which correlated with an increase in biofilm biomass in all isolates. Induction of antibiotic cross-resistance occurred in 6/84 possible combinations of bacteria, biocide, and antibiotic. Relative pathogenicity significantly decreased after triclosan exposure (5/8 isolates), increased after silver nitrate exposure (2/8 isolates), and varied between isolates for PHMB and BAC. The biocompatibility index ranked the antiseptic potential as PHMB > triclosan > BAC > silver nitrate. Biocide exposure in UPEC may lead to reductions in biocide and antibiotic susceptibility, changes in biofilm formation, and alterations in relative pathogenicity. These data indicate the multiple consequences of biocide adaptation that should be considered when selecting an anti-infective catheter-coating agent.

## Abstract (precis)

Uropathogenic Escherichia coli (UPEC) is a frequent cause of catheter-associated urinary tract infection (CAUTI). Biocides have been incorporated into catheter coatings to inhibit bacterial colonization while, ideally, exhibiting low cytotoxicity and mitigating the selection of resistant bacterial populations.

## INTRODUCTION

Catheter-associated urinary tract infections (CAUTI) are among the most commonly acquired health care-associated infections, contributing considerably to patient morbidity and posing an economic burden on health care service providers (1). Complications associated with catheterization often arise due to contamination of the catheter surface with uropathogenic Escherichia coli (UPEC) during catheter insertion, leading to the formation of bacterial biofilms and subsequent infection. Patients undergoing long-term catheterization are at a particular risk of acquiring CAUTI, with studies indicating a 5% to 8% increase in the risk of developing bacteriuria for every day that the catheter remains inserted (2). The majority of patients exhibit bacteriuria after 4 weeks of catheterization, potentially leading to further complications, such as pyelonephritis and septicemia (2, 3).

Bacterial biofilms are often recalcitrant to antimicrobial chemotherapy and to the actions of the host immune system, making biofilm-associated infections, such as CAUTIs, difficult to treat (4). Biofilms show decreased susceptibility to antibiotics, partially due to the shielding effect of the extracellular polymeric substance (EPS) encasing the bacterial cells (5), the low metabolic activity of the cells within the biofilm (6), and the activity of membrane-bound efflux pumps that actively expel antimicrobial compounds from the bacterial cell (4). Furthermore, antibiotic resistance genes are frequently transferred between bacteria within a biofilm by horizontal gene transfer, allowing the dissemination of resistance through a bacterial population (7). Antibiotic treatment of CAUTIs is therefore often ineffective due to the recalcitrance of the biofilm, in addition to the increasing prevalence of antibiotic-resistant uropathogens (8). There is considerable interest in developing anti-infective catheter coatings that are refractory to microbial colonization and subsequent biofilm formation in an attempt to prevent the establishment of CAUTIs.

Biocides are broad-spectrum antimicrobial chemicals that inhibit the growth of or that kill microorganisms (9). Biocide-coated urinary catheters incorporating biocides, such as silver nitrate and nitrofurazone, that are eluted from the surface of the catheter, providing an antimicrobial gradient and a potential selective pressure for biocide-resistant populations of bacteria, have been developed (10). Current clinical trial data have highlighted the limited antimicrobial efficacy of silver-impregnated catheters compared to that of catheters without an antimicrobial coating, while nitrofurazone-containing coatings have been shown to exhibit only short-term antimicrobial activity and may therefore be ineffective in patients undergoing long-term catheterization (11, 12). This has fueled the search for further anti-infective coating agents that display broad-spectrum activity which is maintained after prolonged use.

Long-term exposure of certain bacterial species to biocides may cause the induction of biocide insusceptibility either through the selection of intrinsically resistant mutants or through induced phenotypic adaptations, bringing into question the long-term antimicrobial activity of various biocide-containing coatings (13). Concerns have also been raised that long-term biocide exposure may promote cross-resistance to antibiotics through the acquisition of mutations in shared target sites or through the activation of broad-range defense mechanisms (14), such as increased cellular efflux activity (15) or decreased cell permeability (16). It can, however, be argued that while long-term biocide exposure may lead to reductions in biocide or antibiotic susceptibility in bacteria, these reductions are small and would not impact the susceptibility of bacteria to the concentrations of biocide used in practice. Furthermore, such changes in biocide susceptibility may be accompanied by functional deficits impacting biofilm formation, pathogenicity, and competitive fitness in bacteria (17). Therefore, in order to develop an effective anti-infective catheter coating, the multiple long-term effects of the biocide used within the coating must be taken into consideration.

While previous investigations have evaluated the impact of long-term biocide exposure on the antimicrobial susceptibility of many clinically relevant bacteria, there is no current investigation into the multiple phenotypic consequences that may occur due to long-term biocide exposure in UPEC. The current study therefore aimed to quantify the effects of long-term biocide exposure in eight UPEC isolates. The commonly used biocides polyhexamethylene biguanide (PHMB), triclosan, benzalkonium chloride (BAC), and silver nitrate were evaluated for their long-term antibacterial and antibiofilm activity and their potential to induce antibiotic cross-resistance. The impact that biocide exposure has on bacterial relative pathogenicity was assessed using a Galleria mellonella waxworm model, and the biocides’ antiseptic potential was determined by calculating cytotoxicity in an L929 murine fibroblast cell line, allowing the determination of a biocompatibility index (BI) value (18).

## RESULTS

### Biocide susceptibility of UPEC in planktonic and biofilm states.

MICs, minimum bactericidal concentrations (MBCs), and minimum biofilm eradication concentrations (MBECs) were determined for all test isolates before (at passage 0 [P0]) and after repeated passage either in the absence of a specific biocide (after 12 passages in a biocide-free environment [C12]) or in the presence of a specific biocide (after 12 passages in the presence of each biocide [P12]) (Tables 1 to 3). The change in biocide susceptibility after exposure was calculated as the fold change relative to the susceptibility of the control (C12; see Table S1 in the supplemental material). The data indicate both the frequency of the susceptibility change (≥2-fold) and the average magnitude of the susceptibility change for each biocide.

**TABLE 1:** MICs for UPEC before and after biocide exposure

Isolate	MIC^*a*^ (μg/ml)											
	PHMB			Triclosan			BAC			Silver nitrate		
	P0	P12	C12	P0	P12	C12	P0	P12	C12	P0	P12	C12
EC1	0.5	0.2	0.5	0.00001	2	0.02 (0.01)	15.6	15.6	15.6	31.3	62.5	31.3
EC2	0.2	0.2	0.2	0.1	15.6	0.05 (0.02)	15.6	31.3	15.6	31.3	62.5	31.3
EC11	0.2	0.2	0.2	0.1	2	0.05 (0.02)	15.6	31.3	15.6	31.3	62.5	31.3
EC26	0.5	0.2	0.5	0.2	125	0.03	15.6	31.3	15.6	31.3	62.5	31.3
EC28	0.5	0.5	0.5	0.2	3.9	0.2 (0.06)	15.6	15.6	15.6	31.3	62.5	31.3
EC34	0.2	0.2	0.2	0.03	15.6	0.02	15.6	15.6	15.6	31.3	62.5	31.3
EC958	1	0.2	1	0.1	7.8	0.03	15.6	31.3	15.6	31.3	62.5	31.3
CFT073	1	0.2	1	0.1	15.6	0.02	15.6	15.6	15.6	31.3	31.3	15.6

a MICs are for the UPEC isolates before exposure to biocide (P0), after 12 passages in the presence of each biocide (P12), and after 12 passages in a biocide-free environment (C12). Data represent the mean MICs taken from two separate experiments each with four technical replicates. Data in parentheses represent SDs.

**TABLE 2:** MBCs for UPEC before and after biocide exposure

Isolate	MBC^*a*^ (μg/ml)											
	PHMB			Triclosan			BAC			Silver nitrate		
	P0	P12	C12	P0	P12	C12	P0	P12	C12	P0	P12	C12
EC1	1	0.5	0.7 (0.3)	0.002	7.8	7.8	15.6	31.3	15.6	31.3	62.5	31.3
EC2	1	0.5	1	7.8	31.3	7.8	31.3	31.3	15.6	31.3	62.5	31.3
EC11	1	0.5	0.5	7.8	7.8	7.8	15.6	31.3	15.6	31.3	62.5	31.3
EC26	0.5	0.5	0.5	7.8	125	7.8	62.5	31.3	15.6	31.3	62.5	31.3
EC28	1	1	1	7.8	7.8	7.8	31.3	15.6	19.5 (8)	31.3	62.5	31.3
EC34	1	0.5	0.7 (0.3)	7.8	62.5	7.8	15.6	15.6	15.6	31.3	62.5	31.3
EC958	2	1	1.1 (0.5)	7.8	62.5	7.8	62.5	15.6	15.6	31.3	500	31.3
CFT073	15.6	1	1.1 (0.5)	7.8	31.3	7.8	15.6	15.6	15.6	31.3	31.3	15.6

a Minimum bactericidal concentrations for UPEC before exposure to biocide (P0), after 12 passages in the presence of each biocide (P12), and after 12 passages in a biocide-free environment (C12). Data represent the mean MBCs taken from two separate experiments each with four technical replicates. Data in parentheses represent SDs.

**TABLE 3:** MBECs for UPEC before and after biocide exposure

Isolate	MBEC^*a*^ (μg/ml)											
	PHMB			Triclosan			BAC			Silver nitrate		
	P0	P12	C12	P0	P12	C12	P0	P12	C12	P0	P12	C12
EC1	31.3	2,000	93.8 (36)	7.8	31.3	0.5	250	500	125	2,000	3,000	3,000
EC2	31.3	2,000	93.8 (36)	3.9	250	2	125	500	62.5	3,000	3,000	3,000
EC11	31.3	250	7.8	2	125	0.06	125	125	13.7 (4)	3,000	3,000	54.7 (16)
EC26	31.3	500	78.1 (31)	1	5,000	2	250	250	93.8 (36)	2,500	3,000	3,000
EC28	62.5	2,000	62.5	3.9	125	7.8	125	125	125	4,000	3,000	2,750 (500)
EC34	15.6	500	7.8	1	250	0.2 (0.07)	62.5	62.5	11.7 (5)	3,000	3,000	1,750 (975)
EC958	62.5	1,000	23.5 (9)	7.8	125	1	250	250	62.5	4,000	4,000	3,000
CFT073	31.3	500	35.2 (20)	2	500	1	62.5	250	62.5	2,000	500	1,500 (577)

a Minimum biofilm eradication concentrations for UPEC before exposure to biocide (P0), after 12 passages in the presence of each biocide (P12), and after 12 passages in a biocide-free environment (C12). Data represent the mean MBECs taken from two separate experiments each with four technical replicates. Data in parentheses represent SDs.

In terms of the MIC, after repeated biocide exposure there was a ≥2-fold increase in 4/8 isolates for BAC, 8/8 for silver nitrate, and 8/8 for triclosan compared to that for the respective bacteria passaged in a biocide-free environment (Table 1). In contrast, 4/8 isolates showed a ≥2-fold decrease in MIC after exposure to PHMB. The average fold change in the MIC (between C12 and P12) across the test panel of UPEC isolates was 1.5 for BAC, 0.7 for PHMB, 2 for silver nitrate, and 807.1 for triclosan (Table S1). For the MBC in the biocide-exposed isolates (Table 2), there was a ≥2-fold increase in 4/8 isolates after BAC exposure, 8/8 for silver nitrate, and 5/8 for triclosan. In contrast, 1 isolate showed a decrease in the MBC after PHMB exposure. The average fold change in the MBC after biocide exposure was 1.5 for BAC, 0.8 for PHMB, 3.8 for silver nitrate, and 5.4 for triclosan (Table S1). In terms of the MBEC (Table 3), after repeated biocide exposure there was a ≥2-fold increase in 7/8 isolates for BAC, 8/8 for PHMB, and 8/8 for triclosan. Silver nitrate exposure led to an increase in the MBEC in 1 isolate and a decrease in 1 isolate. The average fold change in the MBEC after biocide exposure was 4.5 for BAC, 29.2 for PHMB, 832.7 for triclosan, and 7.8 for silver nitrate (Table S1). We observed a number of changes in the MICs, MBCs, and MBECs after the passage of bacteria solely in a biocide-free environment compared to the values for the unpassaged parent isolate. We did not, however, see any incidence of a control passaged isolate (C12) exhibiting an MIC, MBC, or MBEC significantly higher (*P* < 0.05) than that for the respective biocide-passaged isolate (P12), with the exception of with PHMB, where the biocide-exposed isolates frequently exhibited a lower MIC and MBC than the unexposed parent strain and the susceptibility of the control passaged isolate subsequently matched the susceptibility of the parent strain.

### Impact of biocide exposure on UPEC biofilm formation.

**FIG 1:**
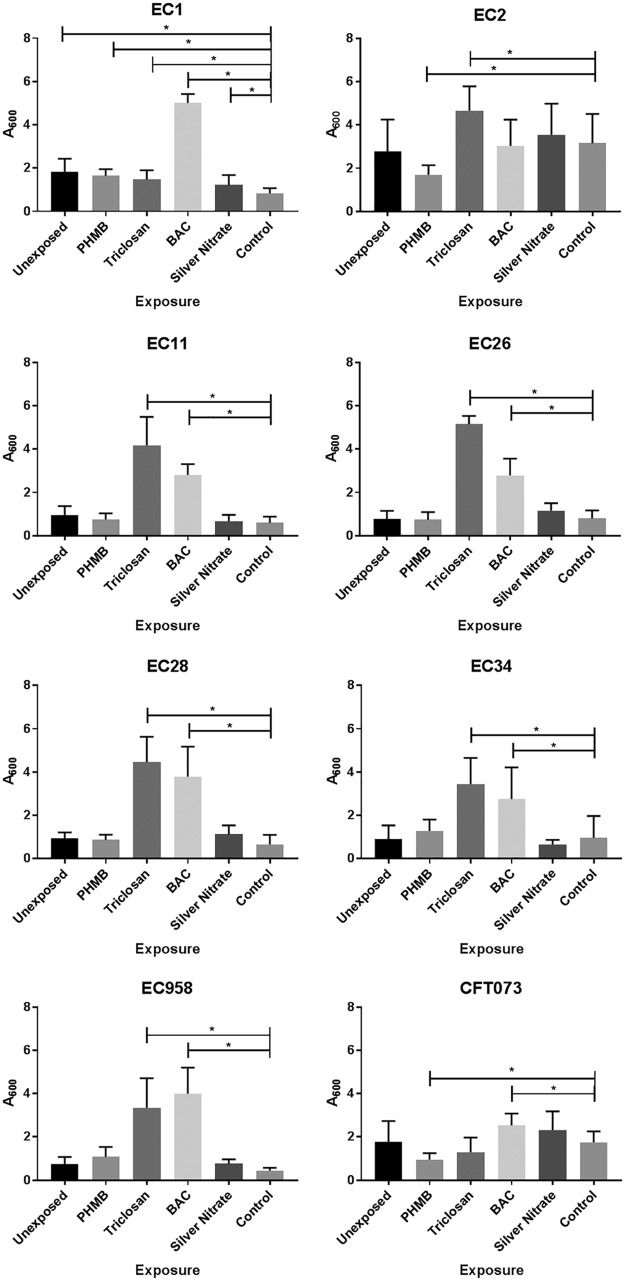
Biofilm formation in biocide-adapted UPEC. The results of a crystal violet biofilm assay indicating the effect of previous biocide exposure on biofilm formation in eight isolates of UPEC are shown. The data show the mean absorbance (*A*_600_), representative of biofilm formation for individual bacteria before and after long-term exposure to PHMB, triclosan, BAC, or silver nitrate or after passage on a biocide-free medium (Control). The data represent those for samples taken from two separate experiments, each with four technical replicates. For data that varied between replicates, SDs are given as error bars. Significance was determined using ANOVA. *, *P* ≤ 0.05.

Biofilm formation was determined via a crystal violet biofilm assay for each UPEC isolate before and after repeated biocide exposure and after passage in a biocide-free medium (Fig. 1). Unexposed isolates displayed various biofilm-forming capabilities prior to biocide exposure, with strain EC2 showing the highest level of biofilm formation, followed by EC1 > CFT073 > EC11 > EC28 > EC34 > EC26 and EC958. When repeatedly exposed to triclosan, all isolates (with the exception of CFT073) demonstrated a significant (analysis of variance [ANOVA], *P* ≤ 0.05) increase in biofilm formation relative to the respective control. All isolates demonstrated a significant increase in biofilm formation after BAC exposure, with the exception of EC2. For PHMB and silver nitrate, EC1 showed a significant increase in biofilm formation after repeated exposure to either biocide. PHMB exposure also induced decreases in biofilm formation in EC2 and CFT073. Differences in biofilm formation were determined to be irrespective of the growth rate, as we did not observe any significant (ANOVA, *P* < 0.05) change in the growth rate or overall growth productivity when in binary culture (Fig. S1).

### Relative pathogenicity of UPEC after long-term biocide exposure.

**FIG 2:**
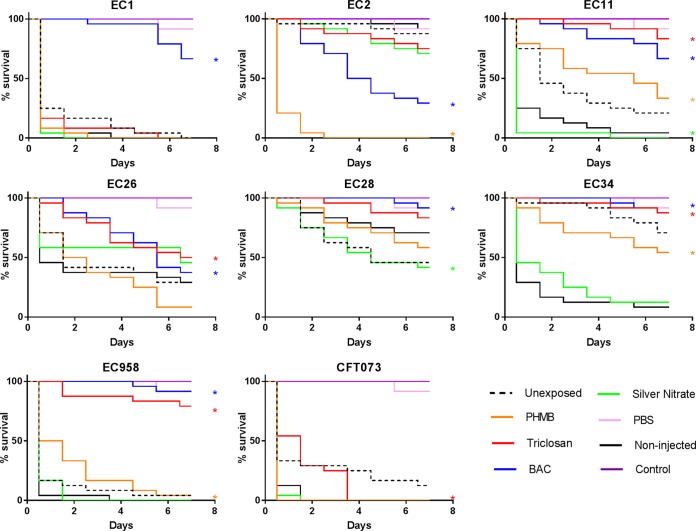
Relative pathogenicity of biocide-adapted UPEC. G. mellonella survival curves for larvae injected with unexposed and biocide-exposed UPEC are shown. Data represent those for 24 biological replicates. Data from noninjected larvae, larvae injected with PBS alone, and larvae injected with control isolates passaged on a biocide-free medium (Control; C12) are also shown. *, a significant difference in pathogenicity when comparing biocide-adapted isolates to the respective control strain (*P* ≤ 0.05, log-rank reduction test).

A G. mellonella waxworm model was used to determine relative pathogenicity in UPEC isolates (Fig. 2). The data indicated that prior to biocide exposure, EC2 was the least pathogenic isolate and that EC1 and EC958 were the most pathogenic isolates. PHMB exposure induced significantly (log-rank test, *P* ≤ 0.05) decreased relative pathogenicity in 3/8 isolates (EC11, EC34, and EC958) and a significant increase in pathogenicity for EC2 compared to that for the respective control isolate (C12). BAC exposure induced significantly decreased pathogenicity in 6/8 isolates (EC1, EC11, EC26, EC28, EC34, and EC958) and significantly increased pathogenicity in EC2. Silver nitrate was the only biocide to induce only significant increases in pathogenicity, which occurred in 2/8 isolates (EC11 and EC28), and triclosan was the only biocide to induce only significant decreases in pathogenicity, which occurred in 5/8 isolates (EC11, EC26, EC34, EC958, and CFT073).  

### Changes in antibiotic susceptibility after biocide exposure.

Isolates were classified as resistant or sensitive to each antibiotic, as defined by British Society for Antimicrobial Chemotherapy (BSAC) breakpoints (19). Antibiotic susceptibility was determined for UPEC isolates before and after exposure to each biocide (Table 4). The data indicate that PHMB exposure induced CFT073 to become resistant to trimethoprim-sulfamethoxazole and EC26 to become resistant to gentamicin. Exposure to triclosan induced nitrofurantoin resistance in EC958 and ciprofloxacin resistance in EC2. Silver nitrate exposure induced EC2 to become resistant to ciprofloxacin, as did BAC exposure. There were cases where isolates that were initially resistant to trimethoprim-sulfamethoxazole became more susceptible after biocide exposure. This occurred in EC2 after exposure to PHMB, BAC, or silver nitrate and in EC11 after exposure to triclosan or BAC. This was also observed for ciprofloxacin in EC11 after triclosan exposure and CFT073 after BAC, triclosan, or silver nitrate exposure.

**TABLE 4:** Antibiotic susceptibility of UPEC before and after biocide exposure

Antibiotic	Exposure	Inhibition zone (mm), susceptibility^*a*^							
		EC1	EC2	EC11	EC26	EC28	EC34	EC958	CFT073
Trimethoprim-sulfamethoxazole	Unexposed	31.8 (1.3), S	0, **R**	0, **R**	0, **R**	0, **R**	0, **R**	0, **R**	30 (0.6), S
	PHMB	31.7 (0.8), S	30.3 (0.6), S	0, **R**	0, **R**	0, **R**	0, **R**	0, **R**	0, **R**
	Triclosan	31.5 (0.6), S	0, **R**	32.3 (0.3), S	0, **R**	0, **R**	0, **R**	0, **R**	28.8 (0.4), S
	BAC	29.7 (3.6), S	26, S	31 (0.6), S	0, **R**	0, **R**	0, **R**	0, **R**	28.8 (1.3), S
	Silver nitrate	32.7 (0.6), S	25.5 (0.5), S	0, **R**	0, **R**	0, **R**	0, **R**	0, **R**	29.5 (0.5), S
Nitrofurantoin	Unexposed	20.3 (0.3), S	20.7 (0.3), S	23.7 (0.3), S	21.2 (1.5), S	19.7 (0.5), S	16.3 (1.2), S	20.4 (1.4), S	18 (0.6), S
	PHMB	20.3 (0.9), S	19.5 (0.5), S	25 (1), S	23.2 (3.1), S	19.2 (0.4), S	15 (0.6), S	19.8 (0.25), S	17.8 (0.8), S
	Triclosan	20 (0.6), S	20.5 (0.6), S	24.7 (0.3), S	24.2 (1.5), S	18.7 (1.4), S	18 (0.6), S	0, **R**	21.2 (0.4), S
	BAC	19.3 (0.3), S	18.5 (0.5), S	23.8 (0.3), S	23.3 (3.1), S	19.5 (1.5), S	15.2 (1.2), S	20.4 (0.1), S	17.2 (0.4), S
	Silver nitrate	20.3 (0.3), S	18.8 (0.3), S	23.5 (1.6), S	23.3 (2.1), S	21.3 (0.5), S	15.8 (0.8), S	20, S	17.2 (0.4), S
Ciprofloxacin	Unexposed	31.2 (0.8), S	34 (0.6), S	13.8 (0.6), **R**	0, **R**	30, S	0, **R**	0, **R**	0, **R**
	PHMB	31.3 (0.8), S	35 (0.6), S	0, **R**	0, **R**	30, S	0, **R**	0, **R**	0, **R**
	Triclosan	32.5 (0.5), S	0, **R**	29.5 (0.8), S	0, **R**	30.7 (1), S	0, **R**	0, **R**	33.2 (1.9), S
	BAC	30 (0.3), S	0, **R**	0, **R**	0, **R**	29.7 (0.8), S	0, **R**	0, **R**	31.2 (1.8), S
	Silver nitrate	31.2 (0.3), S	0, **R**	0, **R**	0, **R**	29.7 (0.8), S	0, **R**	0, **R**	31.7 (2.1), S
Gentamicin	Unexposed	26 (0.5), S	27.7 (0.3), S	25.5 (0.6), S	14.3 (1.2), **I**	18.2 (1), S	16.5 (0.5), **I**	26, S	24.8 (0.4), S
	PHMB	25.5 (0.6), S	28.1 (0.4), S	27.3 (0.3), S	11.8 (0.8), **R**	18.5 (1.6), S	16.2 (1.2), **I**	26.4 (0.5), S	25 S
	Triclosan	25.8 (0.3), S	16, **I**	23.5 (0.6), S	15.8 (3.5), **I**	20.8 (1), S	20 (0.9), S	27 (0.6), S	28.3 (1.4), S
	BAC	26.5 (0.5), S	25.8 (0.8), S	25.3 (0.3), S	16.8 (0.8), **I**	18.8 (0.8), S	18, S	26.7 (0.6), S	24.7 (0.5), S
	Silver nitrate	27.8 (0.3), S	27.2 (0.1), S	27 (0.3), S	15.2 (1.5), **I**	18.7 (0.8), S	17 (1.3), S	26.2 (0.3), S	24, S

a Data show the mean antibiotic inhibition zones for UPEC before and after biocide exposure and represent the results for samples taken from two separate experiments each with three technical replicates. For data that varied between replicates, SDs are given in parentheses. S, sensitive; **I**, intermediate; **R**, Resistant. Susceptibility was defined by use of the BSAC breakpoint (19).

### Biocompatibility index.

Cytotoxicity data for the four biocides against an L929 cell line are shown in Table 5. The concentration of biocide producing a 3-log_10_ reduction (rf), indicating antimicrobial activity, and the corresponding BI values, highlighting the antiseptic potential of the compounds, are shown in Table 6. The order of cytotoxicity in relation to the biocide concentration was silver nitrate > PHMB > BAC > triclosan. The only isolate for which an rf value could be determined for silver nitrate was CFT073, as the rf values for the other isolates exceeded the maximum solubility of the biocide. Similarly, an rf value could not be determined in EC28 and CFT073 for triclosan, as the rf value was greater than the highest achievable test concentration. BI values for the eight isolates were averaged for each biocide, and the final ranked order of BI was PHMB > triclosan > BAC > silver nitrate, indicating the antiseptic potential of the biocides.

**TABLE 5:** Biocide cytotoxicity in an L929 murine fibroblast cell line

Biocide	IC_50_^*a*^ (mg/ml)		Mol wt	Mean IC_50_^*b*^	
	NR	MTT		mg/ml	mmol/ml
PHMB	0.02	0.03	2,800	0.026	0.000009
Triclosan	0.19	0.14	289.54	0.16	0.00057
BAC	0.07	0.03	340	0.047	0.00014
Silver nitrate	0.002	0.003	169.87	0.0027	0.000016

a Mean concentration of biocides allowing 50% survival (IC_50_) of murine fibroblasts after 30 min at 37°C, as determined via NR and MTT assays.

b Mean IC_50_ based on mass and molecular weight. Data indicate the results from two separate experiments each with six replicates.

**TABLE 6:** Concentration of biocide producing a 3-log_10_ reduction for eight isolates of UPEC determined by quantitative suspension test and the resulting BI^*a*^

Biocide	EC1		EC2		EC11		EC26		EC28		EC34		EC958		CFT073	
	rf (mg/liter)	BI	rf (mg/liter)	BI	rf (mg/liter)	BI	rf (mg/liter)	BI	rf (mg/liter)	BI	rf (mg/liter)	BI	rf (mg/liter)	BI	rf (mg/liter)	BI
PHMB	0.02	1.6	0.06	0.4	0.01	1.6	0.02	1.6	0.3	0.1	0.1	0.2	0.5	0.05	0.02	1.6
Triclosan	0.2	0.7	1.1	0.1	0.2	0.7	1.1	0.1	NC	NC	0.6	0.3	2.3	0.07	NC	NC
BAC	0.07	0.7	0.2	0.2	0.2	0.2	0.2	0.2	0.1	0.3	0.2	0.2	0.6	0.08	0.07	0.7
Silver nitrate	NC	NC	NC	NC	NC	NC	NC	NC	NC	NC	NC	NC	NC	NC	0.01	0.2

a Data show the concentration of biocide producing a 3-log_10_ reduction (rf) after 30 min of exposure at 37°C for eight isolates of UPEC and the resulting BI value. NC, not calculable because for certain combinations of biocide and bacterial isolate, the rf value exceeded the maximum solubility of the biocide. The data represent the mean rf values taken from two separate experiments each with four technical replicates.

### Mutation rate frequency in UPEC isolates.

The mutation rate frequency was determined with regards to rifampin resistance. We observed rifampin-resistant mutants of all UPEC isolates (Table 7). Mutation frequencies varied from 1.7 × 10^−8^ for CFT073 up to 3 × 10^−7^ for EC2, with an overall mutation frequency rank order of EC2 > EC28 > EC11 > EC1 > EC34 > EC958 > EC26 > CFT073.

**TABLE 7:** Mutation rate frequencies for unexposed UPEC

Isolate	Mutation rate frequency^*a*^
EC1	8 × 10^−8^ (1.7 × 10^−8^)
EC2	3 × 10^−7^ (1 × 10^−7^)
EC11	1.4 × 10^−7^ (0.4 × 10^−7^)
EC26	3.4 × 10^−8^ (0.3 × 10^−8^)
EC28	1.9 × 10^−7^ (1 × 10^−7^)
EC34	6.8 × 10^−8^ (5.4 × 10^−8^)
EC958	3.5 × 10^−8^ (2.3 × 10^−8^)
CFT073	1.7 × 10^−8^ (1 × 10^−8^)

a Mutation rate frequencies in UPEC isolates resulting in rifampin resistance. Standard deviations are shown in the parentheses (*n* = 3).

## DISCUSSION

The current investigation aimed to explore the phenotypic changes that occur in genetically mixed populations of UPEC as a result of long-term biocide exposure. The susceptibility of eight UPEC isolates in planktonic and biofilm states to a panel of test biocides was determined before and after long-term biocide exposure. The changes to biofilm formation, relative pathogenicity, and antibiotic susceptibility resulting from biocide exposure were assessed. Furthermore, cytotoxicity and the corresponding BI values against an L929 murine fibroblast cell line, indicating the antiseptic potential of the test agents, were determined for each biocide.

### Biocide exposure induces changes in antimicrobial susceptibility in planktonic UPEC.

The data in this investigation highlight that long-term exposure to biocides may influence biocide susceptibility in UPEC. Bacterial susceptibility to biocides can be markedly affected by structural variations in the bacterial cell that (i) impact the attraction of the biocide to the cell (16), (ii) lead to changes in cell permeability to the biocide (20), and (iii) cause a modification in efflux activity, allowing the bacteria to expel the biocide from the cell (21). These modifications may account for some of the changes in biocide susceptibility observed in the current study; however, the exact mechanisms that govern each specific adaptation depend upon a multitude of factors inherent to both the particular biocide and the bacterium (16). Furthermore, previous studies have indicated that biocide exposure in bacteria may result in reversible phenotypic adaptations that occur as a consequence of temporary changes in gene expression, for instance, the induction of stress responses (22). In contrast, other investigations highlight that biocide exposure may lead to the selection of biocide-resistant mutants with stable phenotypes that do not revert in the absence of the biocide (23). This may reflect diversity within the mechanisms of action of biocides, particularly with regards to target site specificity. Bacterial exposure to target site-specific biocides, such as triclosan, appears to readily lead to the selection of mutations in the target enzyme FabI (23), while induced insusceptibility toward membrane-active compounds, such as biguanides (PHMB) and quaternary ammonium compounds (BAC), is often associated with the induction of stress responses (24, 25).

In terms of initial antimicrobial efficacy, silver nitrate demonstrated the lowest activity against planktonic UPEC compared to the activities of the other test biocides at the MICs and MBCs. We observed a high frequency of small-magnitude decreases (≤2-fold) in silver nitrate susceptibility after long-term exposure, resulting in comparatively high MIC and MBC values. Silver is widely considered an effective anti-infective urinary catheter-coating agent and is used in currently marketed anti-infective urinary catheters (26). However, previous investigations have also documented the selection of silver resistance in Gram-negative pathogens (27), including E. coli and other invasive *Enterobacteriaceae* (28). This resistance has been correlated with increased efflux activity (29) or a loss of outer membrane porins (30), thereby decreasing cell permeability, which may explain the induced reductions in silver nitrate susceptibility observed in our UPEC isolates.

PHMB exposure induced a high frequency of small-magnitude (≤2-fold) increases in susceptibility in planktonic UPEC at the MIC and MBC. Previous data indicated that changes in bacterial susceptibility in response to membrane-active compounds, such as biguanides, are usually attributed to alterations in the structural integrity of the bacterial cell envelope, impacting cell permeability; modifications in the structure of lipopolysaccharide (LPS), interfering with electrostatic interactions between the cationic biocide and cell envelope; and increased cellular efflux activity, expelling the biocide from the cell (22). These mechanisms of resistance are in contrast to the data obtained in the current investigation. While other studies have also highlighted increases in PHMB susceptibility in bacteria after long-term exposure, the underlying mechanisms that govern this adaptation remain unknown. It has been suggested that long-term exposure to certain biocides in bacteria may result in cumulative cellular damage and a resulting loss of fitness, increasing bacterial susceptibility over time (13). The potential for PHMB to lead to increased susceptibility in bacteria after long-term exposure is an attractive attribute when considering an antimicrobial catheter-coating agent, particularly in catheters that would be required for longer-term use and that are therefore prone to the selection of resistant microorganisms.

Triclosan was the most potent antimicrobial before repeated biocide exposure in planktonic UPEC. However, triclosan induced the largest frequency and magnitude of susceptibility decreases, according to the MICs and MBCs. The resistance of E. coli to triclosan has been widely documented and is believed to be due to a mutation in the target enzyme, FabI (23), due to increased cellular efflux (15) and changes in the cell membrane composition that reduce permeability (31). Triclosan-impregnated catheters have demonstrated marked efficacy in *in vitro* studies (32) and show little reduction in antimicrobial activity even after long-term use (33). This may be due to the fact that while large susceptibility changes may occur in bacteria following triclosan exposure, as indicated in our data, the initial potency of triclosan means that the catheter maintains a high level of antimicrobial activity even after the bacteria adapt to the presence of the biocide, likely due to its multiple-target-site mode of action.

BAC demonstrated lower initial antimicrobial activity against planktonic UPEC than triclosan and PHMB (according to the MICs and MBCs) and induced only minor reductions (≤2-fold) in susceptibility after long-term exposure. Changes in gene expression in BAC-adapted E. coli have been previously identified, revealing the upregulation of the efflux pump membrane transporter *yhiV* and the downregulation of the outer membrane porin *ompA*, thereby increasing the cellular efflux of BAC and reducing cell permeability toward the biocide (34).

Repeated passage of bacteria on a biocide-free medium occasionally led to changes in biocide susceptibility within the planktonic culture; however, these changes occurred at a substantially lower magnitude and frequency than those observed after biocide adaptation and were predominantly increases in susceptibility. This potentially emphasizes the fitness costs associated with repeated culture. Significantly, we did not see any reduction in biocide susceptibility when the isolate passaged in the absence of biocide was compared to the unexposed parent strain.

### Biofilm formation and susceptibility in UPEC after biocide exposure.

Bacteria that have adapted to the presence of biocides may exhibit further phenotypic alterations, such as changes in growth rate, biofilm formation, and competitive fitness, which may influence pathogenicity (13, 17). After biocide exposure, several UPEC isolates in the current study exhibited significant changes in biofilm formation. While biofilm formation is a complex multifactorial process, these changes could potentially be attributed to the selection of mutants with alterations in factors involved in the establishment of biofilms, such as adhesion, EPS production, or maturation.

Biocide exposure largely led to increases in biofilm formation, particularly after exposure to BAC and triclosan. Of the 7 UPEC isolates that demonstrated an increase in biofilm formation after BAC exposure, 6 had a corresponding increase in MBECs. All 7 isolates that had increased biofilm formation after triclosan exposure also exhibited an elevation in MBECs. PHMB exposure led to a significant decrease in biofilm formation for strains EC2 and CFT073 which did not correspond with decreases in their MBECs, possibly indicating the recalcitrance of persister populations within the biofilm irrespective of biofilm biomass (35).

BAC adaptation has previously been correlated with an increase in biofilm biomass in E. coli, which is believed to be due to an increase in protein and polysaccharide content within the extracellular polymeric substance (EPS) (36). This change in EPS composition may lead to reduced BAC susceptibility, as observed in our BAC-adapted isolates. Yu et al. (37) utilized a genome-wide enrichment screen to demonstrate the genes involved in triclosan adaptation in E. coli. Microarray analysis revealed that triclosan exposure resulted in an increase in the expression of *fimDFHI*, which encodes proteins involved in fimbrial biosynthesis, which has been shown to be positively associated with an increase in biofilm formation (38). This may provide a potential link between the increase in biofilm formation and, thus, the resistance caused by triclosan exposure in the UPEC isolates used in the current investigation.

### Changes in antibiotic susceptibility after biocide exposure in UPEC.

Concerns have been raised that biocide exposure may induce cross-resistance to clinically relevant antibiotics. In the current study, we observed the generation of antibiotic resistance in 6 out of a possible 84 combinations of bacteria, biocides, and antibiotics. The biocides that induced the highest number of cases of cross-resistance in a previously susceptible or intermediate isolate were triclosan, which induced cross-resistance to nitrofurantoin and ciprofloxacin, and PHMB, which induced cross-resistance to trimethoprim-sulfamethoxazole and ciprofloxacin. BAC and silver nitrate exposure led to one observed case of cross-resistance, each of which was toward ciprofloxacin.

There have been previous reports of efflux-mediated cross-resistance between antibiotics and triclosan reportedly due to the upregulation of *acrAB*, encoding the AcrAB efflux pump (39). Efflux pumps have also been correlated with the cross-resistance observed between quaternary ammonium compounds and antibiotics in E. coli. Bore et al. observed reduced antibiotic susceptibility in BAC-adapted E. coli which also coincided with an increase in the expression of *acrAB* and the downregulation of multiple outer membrane porins, including OmpA, OmpF, and OmpT (34). While there is relatively sparse evidence on the generation of antibiotic cross-resistance due to PHMB exposure in bacteria, the mechanisms of uptake of PHMB are similar to those of aminoglycosides and involve destabilization of the bacterial cell membrane and LPS reorganization (40). The interaction between LPS and PHMB is known to be a key step in the initial interaction of the biocide with the bacterial cell in E. coli (26). This may suggest why an induced reduction in PHMB susceptibility in our UPEC isolates also led to a similar reduction in susceptibility to gentamicin. Studies on silver resistance in E. coli have revealed acquired low-level cross-resistance to cephalosporins, similarly due to increased efflux and reduced porin expression (30). In this study, there were 9 cases of biocide exposure eliciting increased susceptibility to trimethoprim-sulfamethoxazole or ciprofloxacin. This occurred in 3 isolates after exposure to BAC or PHMB, in 2 isolates after exposure to silver nitrate, and in 1 isolate after exposure to PHMB. This display of cross-protection has been noted in previous studies and has been suggested to be due to a potential increase in cell permeability in response to biocide adaptation; however, the underlying mechanisms remain unclear (17). An increase in susceptibility to clinically relevant antibiotics in previously resistant uropathogens would be an extremely beneficial attribute when considering a coating agent to combat the establishment of CAUTIs.

### Biocompatibility of test biocides in an L929 cell line.

To assess the suitability of an antiseptic agent, both the antimicrobial activity and cytotoxicity must be considered. Silver nitrate showed the highest level of cytotoxicity in an L929 cell line and the lowest antimicrobial efficacy in the corresponding quantitative suspension test (rf value). The reduced activity of silver in the presence of serum has previously been attributed to the binding of the silver cations to the electronegative serum components, which may explain the low level of antimicrobial activity of silver nitrate observed in the quantitative suspension test in the current study (41). Silver ions have been demonstrated to interact with components of mammalian cells, including mitochondria, nuclei, the endoplasmic reticulum, and the cell membrane (42). The interaction of silver ions with mitochondria reportedly causes mitochondrial damage and the release of reactive oxygen species (ROS), resulting in apoptosis, suggesting a mechanism of silver-mediated cytotoxicity (43). While PHMB was shown to be the second most cytotoxic biocide tested, it exhibited a relatively low rf value, resulting in the highest BI value out of all the test biocides. PHMB has previously shown low-level cytotoxicity toward mammalian cells, including L929 cells, which is suggested to be due to the interaction of the biocide with the mammalian cell membrane, leading to membrane damage (44). BAC was the second least cytotoxic biocide tested and showed the second highest level of antimicrobial activity in the presence of serum in the quantitative suspension tests. BAC has been shown to interact with guanine nucleotide triphosphate-binding proteins (G proteins), impacting cell signaling transduction in mammalian cells and causing DNA damage (45). Cytotoxicity data indicated that triclosan was the least cytotoxic of all the test biocides. However, the rf values were high, resulting in the second highest BI value. Triclosan has previously shown reduced antimicrobial efficacy in the presence of serum; this is believed to be due to the bacterium’s ability to gain an exogenous supply of fatty acids from the serum, thereby bypassing the inhibitory effects of the biocide (46). Additionally, previous studies reported on triclosan interference with mitochondrial respiration (47), in addition to a damaging effect on the plasma membrane and induced apoptotic cell death (48), suggesting a potential mechanism of cytotoxicity.

### Altered relative pathogenicity in biocide-adapted UPEC.

Repeated exposure to the biocide silver nitrate induced an increase in relative pathogenicity in 2/8 isolates of UPEC, while 3/8 PHMB-exposed isolates exhibited a decrease in pathogenicity and 1/8 PHMB-exposed isolates exhibited an increase in pathogenicity. A decrease in pathogenicity was observed after exposure to triclosan in 5/8 isolates and after exposure to BAC in 6/8 isolates. BAC also induced an increase in pathogenicity in 1 further isolate. Triclosan exposure has previously been shown to reduce the relative pathogenicity of certain bacterial species in a G. mellonella waxworm model (49). These pathogenicity changes were suggested to be due to changes in virulence factor production, specifically, reduced DNase activity and the downregulation of cell surface adhesins (50). It has been shown that triclosan exposure specifically downregulates genes encoding the outer membrane proteins P fimbriae and protein X in E. coli (51), which are integral for UPEC attachment to cell surfaces (52) and entry into host cells (53). Isolates of E. coli that have been exposed to BAC have been shown to have increased hemolysin activity and enhanced virulence (54), which may explain the increase in pathogenicity in strain EC2 after BAC exposure. To our knowledge, there are no current studies regarding the effects of silver or PHMB exposure on bacterial virulence factor production and the resulting pathogenicity.

### Consequence of variance in mutation rate frequency in UPEC.

Elevated mutation rates have previously been reported in E. coli strains (55). Furthermore, the adapting populations generated in the current investigation may have led to the selection of hypermutators due to the selective pressures created during biocide exposure. We evaluated the mutation frequencies in our parent isolates to determine whether this correlated with a higher frequency of phenotypic adaptations after biocide exposure. The mutation rate frequency was determined to be ordered EC2 > EC28 > EC11 > EC1 > EC34 > EC958 > EC26 > CFT072. When comparing the mutation rate to the incidences of biocide susceptibility change (MICs, MBCs, and MBECs), EC11 and CFT073 showed the highest frequency of changes in biocide susceptibility, while EC28 showed the lowest. We observed two cases of significant change in biofilm formation for each isolate, with the exception of EC1, for which we observed four. In terms of significant changes in relative pathogenicity, EC11 demonstrated four significant changes after biocide exposure, EC34 and EC958 showed three, EC2, EC26, and EC28 showed two, and EC1 and CFT073 showed one. With regard to changes in antibiotic susceptibility, we saw the highest number of incidences of cross-resistance for EC2. These data indicate a potential correlation when comparing mutation rate frequency and antibiotic cross-resistance in UPEC, but this trend does not extend to all aspects of phenotypic adaptation that occur as a result of biocide exposure.

### Conclusion.

The use of biocides for the purpose of antisepsis has led to concern over the selection of biocide resistance in clinically relevant pathogens. Here we demonstrate that long-term exposure of UPEC to commonly used biocides can result in changes in biocide susceptibility which may be accompanied by further phenotypic alterations impacting biofilm formation, antibiotic susceptibility, and relative pathogenicity. The multiple consequences of bacterial adaptation toward biocides should therefore be evaluated when considering a potential anti-infective catheter-coating agent.

## MATERIALS AND METHODS

### Bacteria and chemicals.

Six UPEC clinical isolates (EC1, EC2, EC11, EC26, EC28, and EC34) previously isolated from urinary tract infections (Stepping Hill Hospital, Stockport, UK) and two laboratory-characterized UPEC strains, EC958 and CFT073, were used in the investigation. Bacteria were cultured on Muller-Hinton agar (MHA; Oxoid, UK) and Muller-Hinton broth (MHB; Oxoid, UK) and incubated aerobically at 37°C for 18 h, unless otherwise stated. Biocides were formulated as follows: triclosan was solubilized in 5% (vol/vol) ethanol. Polyhexamethylene biguanide (PHMB; Lonza, Blackley, UK), benzalkonium chloride (BAC), and silver nitrate were prepared at 1 mg/ml in water and filter sterilized prior to use. All chemicals were purchased from Sigma-Aldrich (Poole, UK), unless otherwise stated.

### Long-term exposure of bacteria to biocides.

Bacteria were repeatedly exposed to biocides using an antimicrobial gradient plating system adapted from that of McBain et al. (56). In brief, 100 µl of a 5× MBC solution of biocide was added to an 8- by 8-mm well in the center of a 90-mm agar plate. Bacterial pure cultures were radially inoculated in duplicate from the edge of the plate to the center, prior to incubation for 2 days aerobically at 37°C. The biomass from the inner edge of the annulus of bacterial growth, representative of the highest biocide concentration at which growth could occur, was removed and used to inoculate a new biocide-containing plate, as outlined above. This process was repeated for 12 passages. Control isolates passaged 12 times on biocide-free medium were also included. Bacteria were archived at −80°C before and after biocide passage for subsequent testing.

### MIC and MBC.

The MIC and minimum bactericidal concentration (MBC) were determined as described previously (17). In brief, two 5-ml overnight cultures of test bacteria were prepared in MHB prior to overnight incubation (18 to 24 h) at 37°C and 100 rpm. Cultures were diluted to an optical density at 600 nm (OD_600_) of 0.008 in 20 ml of sterile MHB to produce a bacterial inoculum for biocide susceptibility testing. Doubling dilutions (150 µl) of each test biocide were prepared in sterile MHB in a 96-well microtiter plate prior to addition of the bacterial inoculum (150 µl). The plates were incubated overnight (18 to 24 h) at 37°C and 100 rpm. The MIC was defined as the lowest concentration of biocide at which growth was completely inhibited (viewed as turbidity relative to that for a sterile negative control). To determine the MBC, aliquots (5 µl) were taken from the wells of the MIC plate and were spot plated onto Mueller-Hinton agar (MHA) in triplicate. The plates were incubated statically for 18 to 24 h at 37°C. The lowest test concentration at which visible bacterial growth was completely inhibited was deemed the MBC.

### Minimum biofilm eradication concentration.

Minimum biofilm eradication concentrations were determined using a Calgary biofilm device (CBD) as described previously (57). Briefly, two 5-ml overnight cultures of test bacteria were prepared in MHB and were incubated for 18 to 24 h at 37°C and 100 rpm before being diluted to an OD_600_ of 0.008 in MHB to create a bacterial inoculum for biofilm susceptibility testing. One hundred microliters of bacterial inoculum was added to each well of the CBD base, and the plates were incubated at 37°C for 48 h to allow biofilm formation on the pegs. Doubling dilutions of biocides were prepared in sterile broth across a 96-well microtiter plate. Biofilms were exposed to antimicrobial compounds and incubated for 24 h at 37°C and 100 rpm. After incubation, the pegged lid was transferred to a 96-well plate containing 200 μl of sterile broth and was incubated for 24 h at 37°C and 100 rpm. The MBEC was defined as the lowest concentration of biocide at which regrowth was completely inhibited (viewed as the turbidity relative to that for a sterile negative control), indicating complete biofilm eradication.

### Crystal violet bacterial attachment assay.

Two 5-ml overnight cultures of test bacteria were diluted to an OD_600_ of 0.008 in MHB after incubation for 18 to 24 h at 37°C and 100 rpm. One hundred fifty microliters of diluted overnight bacterial culture was added to the wells of a sterile 96-well microtiter plate. The plates were incubated statically for 48 h at 37°C. The medium was removed from the wells and replaced with 180 µl of crystal violet solution. The plate was left at room temperature for 30 min, the crystal violet solution was decanted, and the wells were rinsed three times with 200 µl of phosphate-buffered (PBS) each time, prior to drying for 1 h at 37°C. The remaining crystal violet was solubilized in 250 µl of 100% ethanol. The *A*_600_ of the solubilized crystal violet solution was determined and compared to that for a sterile MHB negative control.

### Galleria mellonella pathogenicity assay.

The pathogenesis model was adapted from that of Peleg et al. (58). Final larval-stage G. mellonella waxworms (Live Foods Direct, Sheffield, UK) were stored in the dark at 4°C for up to 7 days, before randomly assigning 24 to each treatment group and incubating the waxworms at 37°C for 30 min. Overnight suspensions of E. coli were pelleted via centrifugation at 13,000 rpm, washed twice in 1 ml of PBS, and then diluted appropriately to achieve an OD_600_ of 0.1 (5 × 10^5^ to 8 × 10^5^ CFU/ml, as confirmed by colony counts on MHA). Aliquots of each suspension (5 µl) were injected into the hemocele of each larva via the last left proleg using a Hamilton syringe. Larvae were incubated in a petri dish at 37°C, and the number of surviving individuals was recorded daily. An untreated group and a group injected with sterile PBS were used as additional controls. The experiment was terminated when at least two individuals in a control group had died or after 7 days of incubation. Two independent bacterial replicates were used to inoculate 24 caterpillars (12 per replicate), and the significance of the death rate was calculated using a log-rank reduction test (*P* ≤ 0.01).

### Biocompatibility index.

Calculation of the biocompatibility index (BI) was performed as described by Muller and Kramer (18). To determine cytotoxicity, neutral red (NR; 3-amino-7-dimethylamino-2-methylphenazine hydrochloride) assays and 3-(4,5-dimethylthiazol-2-yl)-2,5-diphenyltetrazolium bromide (MTT) assays were performed on an L929 cell line to establish the 50% inhibitory concentration (IC_50_). Procedures for the NR assay and the MTT test have been described in detail elsewhere (18). The bacterial quantitative suspension tests were done in accordance with the guidelines for testing disinfectants and antiseptics of the European Committee for Standardization (59). Suspension tests were performed in the presence of serum to determine the rf value, defined as the concentration of biocide that achieved a reduction in the bacterial load of at least 3 log_10_ (99.9%). Suspension tests were conducted as follows: overnight bacterial cultures were diluted to 10^8^ to 10^9^ CFU/ml, as determined by colony counts on MHA. Fifteen-microliter aliquots of inoculum were then transferred into 135 µl of biocide-containing cell culture medium prior to incubation for 30 min at 37°C. For PHMB, BAC, and triclosan, the biocide was subsequently inactivated by transfer of 15 µl of the suspension into 135 µl of TSHC (3% [wt/vol] Tween 80, 3% [wt/vol] saponin, 0.1% [wt/vol] histidine, 0.1% [wt/vol] cysteine). Silver nitrate was inactivated using TLA-thio (3% [wt/vol] Tween 80, 0.3% lecithin from soy bean, 0.1% [wt/vol] histidine, 0.5% [wt/vol] sodium thiosulfate). After 30 min of inactivation, 5-µl aliquots were spot plated onto MHA in triplicate. The plates were incubated statically for 18 to 24 h at 37°C, and the number of CFU per milliliter was determined. The lowest test concentration which achieved at least a 3-log_10_ (99.9%) reduction in bacterial load was deemed the rf value. BI was calculated as IC_50_/rf for each combination of biocide and isolate and indicates the antiseptic potential of the test compound.

### Antibiotic susceptibility.

Bacterial susceptibility to trimethoprim-sulfamethoxazole (25 µg), nitrofurantoin (50 µg), ciprofloxacin (10 µg), and gentamicin (200 µg) was determined. Antibiotic susceptibility tests were performed according to the standardized British Society for Antimicrobial Chemotherapy (BSAC) disc diffusion method for antimicrobial susceptibility testing (19).

### Determination of mutation rate frequency.

The mutation rate frequency was determined as described by Miller et al. (60) In brief, 100-µl aliquots of a diluted overnight culture obtained from single bacterial colonies were plated onto antibiotic-free MHA plates and MHA plates containing 50 µg/ml rifampin in triplicate. The plates were incubated for 24 h at 37°C, prior to determination of the viable count. Mutation frequencies were expressed as the number of resistant mutants recovered as a fraction of the total number of viable bacteria.

## Supplementary Material

Supplemental file 1
