# Supplementary material for: Biocide Exposure Induces Changes in Susceptibility, Pathogenicity, and Biofilm Formation in Uropathogenic Escherichia coli
Source: Antimicrob Agents Chemother. 2019 Feb 26;63(3):e01892-18. doi: 10.1128/AAC.01892-18 (PMC6395906; doi:10.1128/AAC.01892-18)
Supplement: Supplemental file 1 [file AAC.01892-18-s0001.pdf]

Table S1: Average fold-change in biocide susceptibility for UPEC after repeated biocide exposure.

	**MIC**				**MBC**				**MBEC**			
**Isolate**	**PHMB**	**Triclosan**	**BAC**	**Silver nitrate**	**PHMB**	**Triclosan**	**BAC**	**Silver nitrate**	**PHMB**	**Triclosan**	**BAC**	**Silver nitrate**
**EC1**	**0.5**	**97.7**	1	**2**	0.7	1	**2**	**2**	**21.3**	**62.6**	**2**	1
**EC2**	1	**312.5**	**2**	**2**	**0.5**	**4**	**2**	**2**	**21.3**	**125**	**8**	1
**EC11**	1	**39.1**	**2**	**2**	1	1	**2**	**2**	**32**	**2083.3**	**9.1**	**54.8**
**EC26**	**0.5**	**4166.7**	**2**	**2**	1	**16**	**2**	**2**	**6.4**	**2500**	**2.7**	1
**EC28**	1	**19.5**	1	**2**	1	1	0.8	**2**	**32**	**16**	1	1.1
**EC34**	1	**781.3**	1	**2**	0.7	**8**	1	**2**	**64**	**1250**	**5.3**	1.7
**EC958**	**0.25**	**260**	**2**	**2**	0.9	**8**	1	**16**	**42.5**	**125**	**4**	1.3
**CFT073**	**0.25**	**780**	1	**2**	0.9	**4**	1	**2**	**14.2**	**500**	**4**	**0.3**

Fold changes are indicated for MIC, MBC, and MBEC in UPEC isolates after long-term biocide exposure compared with the respective isolate passaged in a biocide free-environment (C12). Changes (≥2 fold-change) are shown in bold.

Figure S1: Planktonic growth in UPEC isolates

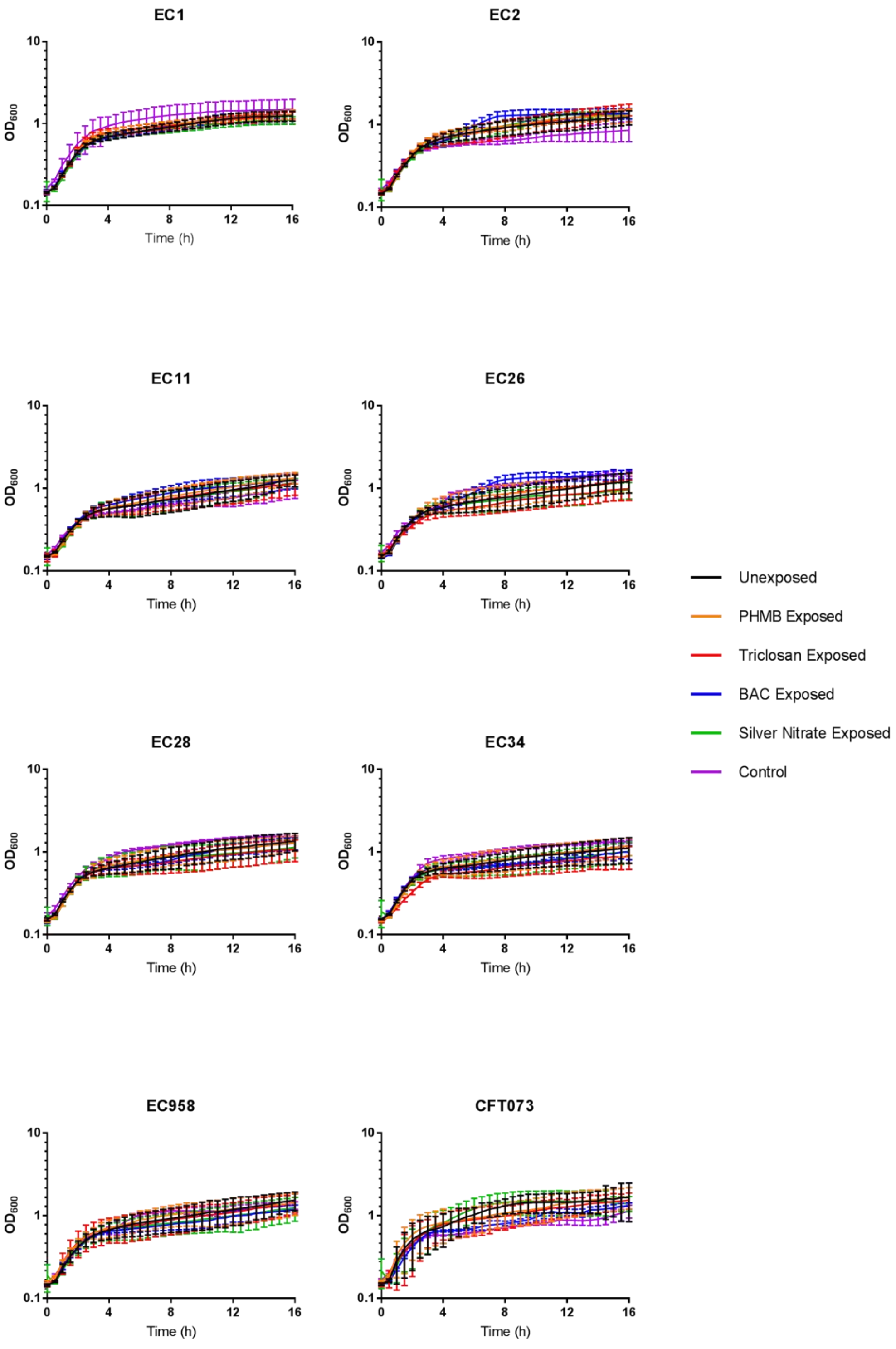

Figure S1: Planktonic growth for eight UPEC isolates after exposure to four biocides. Control isolates repeatedly passaged without biocide (C12) are also shown.
